# Five Fristonian Formulae

**DOI:** 10.3390/e27090944

**Published:** 2025-09-10

**Authors:** Thomas Parr, Giovanni Pezzulo, Rosalyn Moran, Maxwell Ramstead, Axel Constant, Anjali Bhat

**Affiliations:** 1 Nuffield Department of Clinical Neurosciences, University of Oxford, Oxford OX3 9DU, UK; 2 Institute of Cognitive Sciences and Technologies, National Research Council, 00185 Rome, Italy; giovanni.pezzulo@istc.cnr.it; 3 Department of Neuroimaging, Institute of Psychiatry, Psychology and Neuroscience, King’s College, London SE5 8AF, UK; rosalyn.moran@kcl.ac.uk (R.M.); anjali.bhat@kcl.ac.uk (A.B.); 4 Stanhope AI, London SW10 0JG, UK; 5 Noumenal Labs, Dallas, TX 75367, USA; maxwell.d.ramstead@gmail.com; 6 Queen Square Institute of Neurology, University College London, London WC1N 3BG, UK; 7 Department of Engineering and Informatics, The University of Sussex, Brighton BN1 9RH, UK; axel.constant.pruvost@gmail.com

This paper is the contribution of the editorial team for a special issue designed to celebrate the scientific contributions of Karl Friston on his 65th birthday. We have chosen to scaffold this contribution around 5 equations that highlight the unparalleled impact Karl has had upon neuroscience and related fields. These equations form an approximate chronology from the use of simple generative models in statistical inference, through non-linear dynamical systems, leading to an understanding of our brains’ generative models and the ways in which they guide behaviour.

The aim of this editorial, aside from suggesting a verse for the ‘Twelve days of Fristmas,’ is to highlight some of the many contributions Karl Friston has made to our collective understanding of how the brain works and, possibly, how we might build artificial brains. The five equations we have chosen are points along a trajectory through the evolution of neurobiology over the last three decades or so. Starting with the development of methods to understand functional brain imaging data, and ending with a discussion of intelligent behaviour, we will see common themes emerging. These include the role of generative models in neuroscience and beyond, the unification of different formal perspectives of behaviour, and the central importance of (Bayesian model) evidence. Each of these steps will be useful in contextualising the submissions to this special issue.

Our starting point is the use of the general linear model (GLM) in the mass univariate analysis of brain data. This, combined with the use of random field theory [1] to determine appropriate corrections across voxels, underpins the process of Statistical Parametric Mapping (SPM). This approach originated in functional brain mapping using Positron Emission Tomography (PET) imaging [2] and is now widely used for analysis of functional (fMRI) magnetic resonance imaging [3]. There is an elegantly simple idea that underpins SPM—Karl’s original solution to the not-so-simple problem of parsing signal from noisy, multivariate neuroimaging data. The statistical effect of some condition—for example, the presentation of a sensory stimulus—can be computed for each voxel in an image, allowing this effect to be mapped onto the imaged brain to test hypotheses about the functional anatomy of some cognitive or sensorimotor function.

Figure 1 shows a graphic of such a map projected onto an axial slice of a brain, and the equation of the GLM that underwrites this [4]. This linear equation is a generative model—i.e., a probabilistic specification of how some unobserved causal variables influence measurable data. It sets out, in a simplified way the process by which the signal in that voxel is caused by the experimental conditions and interventions that we have imposed. (for the sake of brevity, we will ignore the use of haemodynamic models for deconvolution of the signal in fMRI—e.g., [5]). As we will see, the concept of a generative model is central to all five of the formulae we review here.

A common theme of the work reviewed here is that many familiar procedures can be recovered from a single equation under the right assumptions. When different forms are chosen for the **X** matrix, the resulting estimated ***β*** values, and linear combinations of these, are the statistics used in, for example, *t*-tests or ANOVAs—unpacked in detail in the treatment in [7]. As we will see, one can often derive apparently disparate results by looking at the same equation (here, the GLM) from different perspectives.

The first of the five formulae we have considered represents one of the simplest forms of generative model. The estimation of its parameters is key for several common statistical techniques and can be estimated simply via a least square or a maximum likelihood method. The importance of the GLM paradigm is acknowledged in a contribution to this special issue from Mazor and Mukamel [8]. The authors also highlight the limitations of this sort of model—among which are the restrictions that assumptions of linearity place upon inferences about the non-linear mechanisms by which brain signals are generated. They suggest that one alternative is the use of ‘model free’ methods, giving a detailed and balanced account of a proposition based upon correlative metrics.

As highlighted by Mazor and Mukamel, the GLM, while powerful in its simplicity, does not fully capture the complexity of biological mechanisms. The machinery of maximum likelihood estimation is limited in that it neglects important prior information and sources of uncertainty. While this standard GLM approach has been foundational in the localisation-based analysis of fMRI data, neuroimagers are increasingly building on the simple modular perspective of brain function to embrace a more nuanced, connectivity-based understanding of multi-region networks that underpin information processing in the brain [9]—something that was a core feature of the contribution from Wang, Gonzalez-Martinez [10]. This has been framed as a move from functional segregation to functional integration [11]. These issues lead us to our second Fristonian formula.

For a truly mechanistic understanding of a biological process, we need to account the dynamics (that is, the time evolution) of the underlying physiological processes and the way in which physiological states give rise to measurable data. Dynamic Causal Models (DCMs) [12], an example of which is outlined in Figure 2, are formulated as probabilistic generative models of this physiology. By specifying prior plausibility ranges for the parameters of both the dynamic and observation processes, DCM uses a Bayesian approach, as opposed to the maximum likelihood approach outlined above. More specifically, solutions to DCMs depend upon application of approximate (or variational) Bayes—a set of methods that find tractable approaches to solving apparently intractable inference problems [13]. A central feature of variational inference is that the process of finding good estimates for the posterior probabilities of parameter values (given some data) also estimates the evidence those data afford a particular model [14]. As such, DCM is used for both parameter estimation and for hypothesis testing through model comparison. In neuroimaging, such models often take the form of different network architectures whose (population-level) synaptic rate constants are estimated to determine the network activity that best explains the acquired data.

Figure 2 expresses variational inference as finding the approximate posterior probability *q* that minimises a functional (function of a function) *F*. The *F* here is the free energy objective function of variational inference [13,15,16], being approximately equal to the negative log marginal likelihood (or model evidence) at its minimum. The corresponding *q* that minimises this is the best approximation to an exact posterior probability that can be achieved within the family of distributions to which *q* belongs. This free energy will be central to all subsequent Fristonian formulae considered here. It affords simplification of the procedures outlined in relation to Figure 1, as we now have only one test statistic we ever need consider: the difference in free energy (i.e., log Bayes factor) between the hypotheses we wish to compare.

The graphic in Figure 2, adapted from [17], shows the interaction between different cell types of the neocortex, effectively replicating a canonical microcircuit [18]—a concept we will revisit in relation to Figure 3. This is one of several forms of model that has been used for dynamic causal modelling for fMRI [19], electro- and magnetoencephalography (E/MEG) [20], and even for Bayesian fusion of the two modalities [21]. They are not restricted to cortex and can be applied to ask questions about generic neuroanatomical networks [22]. Such models can be employed in event-based [23] or resting-state data [24], where they can be applied to either timeseries [25] or spectral data [26]. In recent years, the use of DCM has broadened even beyond neuroimaging. Most notably it was applied to questions in epidemiology during the COVID-19 pandemic [27,28,29]. The theme of causal analysis, including in functional imaging data, is picked up in this issue by Sun et al. [30], who outline an approach for directed estimates of effective connectivity from fMRI data and highlight links between their approach, Granger Causality, and DCM (see also [31]).

The third formula we have chosen, in Figure 3, is an expression of a generalised filtering scheme. Generalised filtering [32], with close connections to the extended Kalman-Bucy filter [33], acts as a useful conceptual bridge between an extension of DCMs to stochastic differential equations and an important neurobiological theory—predictive coding. It also provides a point of contact to various control schemes, including proportional-integral-derivative (PID) controllers [34]. From an inference perspective, the use of generalised filtering relies upon the same optimisation of a free energy functional as the process of DCM inversion outlined above but now performs a gradient descent in a moving frame of reference.

The moving reference frame depends upon estimating not just the current state of a system but also its rate of change, the rate of change of the rate of change, and so on. In other words, it is a representation of a local trajectory in terms of a Taylor series expansion around the current time [35], where the position, velocity, acceleration and subsequent coordinates of motion are the coefficients of this Taylor series. This means that when free energy is minimal, and its gradients are zero, our gradient descent should not stop. This is because, if we have estimated a non-zero velocity for our variable of interest, we should continue to move our estimate of the position in line with this velocity even when at the free energy minimum—which itself will move based upon that velocity. A further benefit of this sort of scheme is that it generalises to stochastic systems with coloured (i.e., autocorrelated) noise processes [36]. Such systems are handled by accounting for anticipated covariance between position and acceleration, velocity and jerk, and other pairs of generalised coordinates of motion [37].

**Figure 3:**
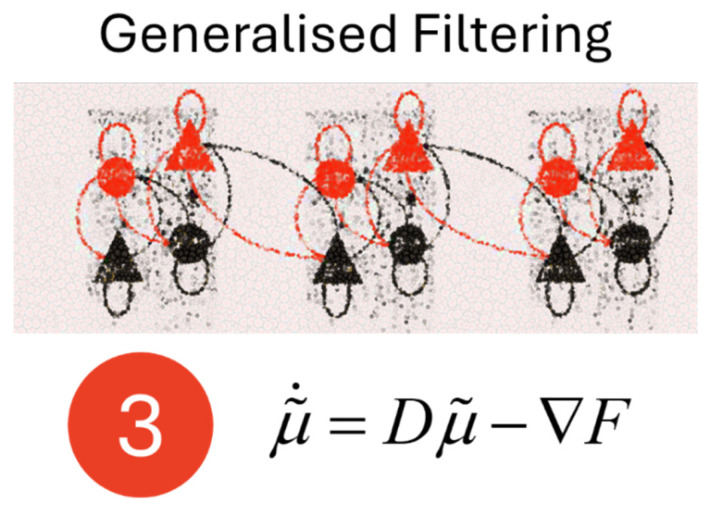
Generalised Filtering. This figure shows the third of our five formulae. This shows a generalised gradient descent on a free energy functional (*F*). The *µ* variables are the modes of the variational (approximate) posterior density over the causes of some data. The tile (~) symbol indicates that we are working with generalised coordinates of motion (i.e., the position, velocity, acceleration, and subsequent orders of motion). The *D* matrix plays the role of a differential operator. In generalised coordinates, this simply means operating on a vector to shift all elements up by one. Under Laplace assumptions, this equation reduces to adjustment of expectations to minimise prediction errors. For hierarchical models, this implies a kind of stereotyped asymmetrical message passing, which closely matches that observed in the anatomical organisation of the neocortex, as highlighted in the graphic, with stereotyped message passing between deep (black) and superficial (red) cell populations. For this reason, generalised filtering provides a conceptual bridge between inference about stochastic timeseries data and predictive coding theories of brain function. The graphic is adapted from those that appear in [38] and related papers.

In addition to its power in analysing timeseries data, generalised filtering provides an important neurobiological advance via its link to predictive coding [39]. Predictive coding is an idea whose early application was in understanding the function of the retina [40] and is based upon the idea that our brains function through a process of making top-down predictions about sensory data, and using prediction errors to adjust our expectations, driving perceptual inference. Of note, the equation for generalised filtering reduces to a form of predictive coding once the free energy is approximately quadratic in the expectations (i.e., a Laplace assumption). Here, we see the use of a method that we might use to analyse data being used as a hypothesis for how our brains might engage in perceptual processing.

Several of the contributions to this special issue are motivated by the notion of predictive coding (or, the broader term, predictive processing). These include Hess et al.’s [41] account of allostatic self-efficacy theory and its roots in interoceptive inference, Medrano and Sajid’s [42] analysis of multi-stable states for predictive coding based upon generative models of chaotic dynamical systems, and Collerton et al.’s [43] focus on the role of ascending and descending messages on the genesis of visual hallucinations. Wright and Bourke [44] consider an interpretation of such messages and error resolution in terms of mesocortical anatomy and Kim [45] unpacks the same sort of dynamics in for synaptic learning—drawing from path-integral formulations of free energy functionals.

Deakin et al. [46] take inspiration from the use of formal computational and mathematical theories, like predictive coding, for perceptual inference and offer an analysis of drift diffusion modelling for perceptual discrimination to complement this. As noted by Xavier et al. [47], the generalisation of predictive coding under the broader principle of free energy minimisation allows one to understand brain dynamics in terms of changes in information theoretic quantities, which the authors explore in the context of the entropy of coupled oscillators.

Interestingly, considering the dual interpretation of Figure 3 as data analysis and neuronal message passing, two of the contributions to this issue deal with computation in neuromorphic architectures. Tucker and Luu [48] question the feasibility of neuronal reconstruction—or the emulation of an individual brain. Hamburg et al. [49] focus on questions of the design of artificial embodied systems and sketch out a way forward, which also draws upon some of the material that follows.

In the above, we have highlighted the role of free energy in inference, both in DCM and in the predictive coding like dynamics of generalised filters. However, it is only with the introduction of the Free Energy Principle (FEP) [50,51] that the profound role that free energy plays in behaviour, sense-making, and self-organization becomes apparent. The FEP is a mathematical statement about the dynamics of systems that interact across a boundary (formally, a ‘Markov blanket’ [52]), a simple instantiation of which is shown in Figure 4. The FEP describes the fundamental imperative—minimizing free energy—of goal-directed behaviour in systems at every scale: organelles, cells, organs, organ systems [53], organisms, societies—anything that can be defined as a bounded, self-organising, entity. It is therefore particularly useful in representing the imperatives of biological systems like the human brain. In the realm of neuroscience, this mathematical statement extends the predictive processing frameworks described above by offering the insight that free energy can be reduced both by changing our posterior beliefs to better fit our sensory data, but also by changing our sensory data (through action) to better fit our internal model of the world. This idea has been particularly influential in the neuroscientific study of movement and agency—where action effectively becomes a process of reflexive fulfilment of predictions—prompting a reformulation of motor commands as proprioceptive predictions [54].

The importance of action is evident in Clark’s [56] conceptual analysis of the way in which the predictive brain can be tricked or ‘hacked’ including the impact of prior beliefs on behaviour as compellingly articulated in the context of Hoover’s sign for functional neurological weakness. Badcock [57] similarly exploits the unified action-perception objective in addressing pathological behaviour from a psychiatric perspective. Neuropsychiatry is well-represented in this issue, with a review and perspective by Ruffini et al. [58] and a view from practical psychotherapy by Holmes [59]. Central to many accounts of pathology under the FEP is the notion of precision [60,61], which determines the relative influence of various prior and conditional distributions in a generative model. While this is important for predictive coding, it has even greater implications for action under the free energy principle, as outlined by Limanowski et al. [62].

The role of the Markov blanket in information exchange prompts an interesting analysis by Fields et al. [63], who draw on quantum information theory to unpack the relationship between informational and thermodynamic energy exchanges. The Markov blanket also plays a central role in the analysis of self-representation in generic physical systems by Fields et al. [64]. The ‘first principles’ approach exemplified by the FEP is part of the motivation for the contribution from Sun et al. [65], which attempts to understand the emergence of patterning along the anterior-posterior axis of the telencephalon by drawing from first principles in dynamical systems. Radomski and Dolega [66] pick up the theme of first principles, with a discussion about the conceptual points of contact, and the gaps between, the FEP and Hamilton’s principle of least action in physics.

Although active inference can be regarded as a direct corollary of the free energy principle, there is an additional move made that has been highly influential and takes us beyond the reflexive account above. This is the idea that planning and decision making can be articulated as model selection problems [67,68], where we choose between alternative models of how the future might play out based upon our actions [69,70]. While free energy, being a function of observations, cannot be used to adjudicate between futures we have yet to observe, we can make use of an expected free energy. This quantity naturally accounts for trade-offs between exploration and exploitation, between risk and ambiguity aversion, and can be used to prospectively score the relative plausibility of different courses of action. The resulting behaviours have the appearance of purpose and, perhaps, sentience [71,72]. As in the graphic of Figure 5, planning as inference can take place as part of a hierarchy [73,74], allowing for multiple timescales to interact in the generation of behaviour.

In this issue, Kiefer [75] provides an interesting account of the intrinsic motivation that follows from this scheme, and its relationship to constrained maximum entropy formulations. Fisher and Hohwy [76] pick up on similar themes, with a focus on the ‘optimism bias’, or implicit belief that our actions will lead to positive outcomes. Nehrer et al. [77] introduce an implementation of active inference principles in the Julia programming language, which is then used by Waade et al. [78] to model the development of shared generative models amongst multiple agents. Multiagent systems are a key theme here, with Albarracin et al. [79] discussing such systems using an approach at the intersection of Husserlian phenomenology and category theory. Paul et al. [80] develop theoretical accounts based upon counterfactual learning in active inference and illustrate these points with numerical simulations. Carl [81] outlines a conceptual approach to the process of translation between languages that appeals to the same underlying computational architectures. Hierarchy in both space and time are central to the model of navigation advanced by de Tinguy et al. [82]. Matsumoto et al. [83] go beyond simulation environments and implement some of these principles in physical robotic systems.

We are grateful for the wide range of fascinating submissions to this special issue, which serve to highlight the broad reach of the topics we have outlined here. Interestingly, it is the equations of Figure 4 and Figure 5 that have prompted the bulk of the submissions to this special issue. This perhaps reflects the current direction of travel for the field, and some of the key opportunities ahead for the growing body of researchers engaged in the Active Inference Framework (AIF). It is difficult to overstate the impact each of the advances outlined in Figure 1, Figure 2, Figure 3, Figure 4 and Figure 5 have had in neuroscience, artificial intelligence, and beyond. Happily (or perhaps crucially), these equations have helped many scientists—including us—to finesse difficult problems and now undergird much of modern theoretical neurobiology. In other words, they are an apt celebration of the unparalleled contributions Karl Friston has made to our understanding of the brain. Having briefly reviewed SPMs, GLMs, DCMs, the FEP, and the AIF, we look forward to Karl’s next great advance, and its associated TLA (three letter acronym). We wish him a (now very belated) Happy Birthday.

## Figures and Tables

**Figure 1:**
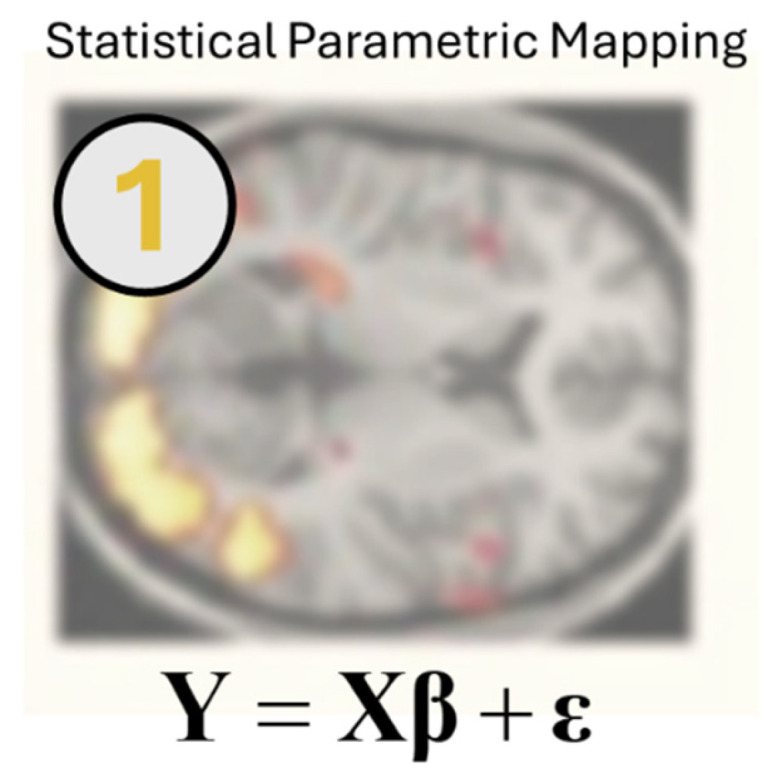
The General Linear Model. This figure shows the first of our five formulae: The general linear model that underwrites much of the statistical parametric mapping methodology used in functional brain imaging. This equation underwrites many of the common statistical procedures we might perform but has a particular role in the mass univariate analysis of brain data. In the equation in this figure, **Y** is an array detailing the signal from each voxel—i.e., with columns representing voxels and rows representing datapoints. The design matrix, **X**, has columns representing different explanatory or confounding variables; and rows representing the datapoints. Each element details the state of the explanatory variables when a given datapoint was measured. The magnitude of the effect of those explanatory variables on the measured signal is given by the **β** matrix, whose rows correspond to the explanatory variables and columns to the voxels. Finally, **ε** is a matrix whose elements are stochastic (i.e., random) variables. Typically, these are assumed to be normally distributed with a mode of zero. The mass univariate approach alluded to above essentially makes the rather strong assumption that the column vectors of **ε** are independent of one another. However, this relatively extreme assumption is corrected for with use of random field theory [1] to set appropriate thresholding during statistical analyses. To generate an image as in Figure 1 (adapted from [6]), we estimate the elements of the **β** matrix for each voxel. These are typically estimated using maximum likelihood or least squares estimation, which involves finding the values of **β** for which the measured **Y** are most probable under the general linear model. The estimated values of **β**—and sometimes linear combinations (statistical contrasts) of their elements—for each column can then be used to calculate statistics that can be rendered onto an image of the brain. These estimates are typically of the form ***β*** = (**X***^T^***X**)^−^**X***^T^***Y**.

**Figure 2:**
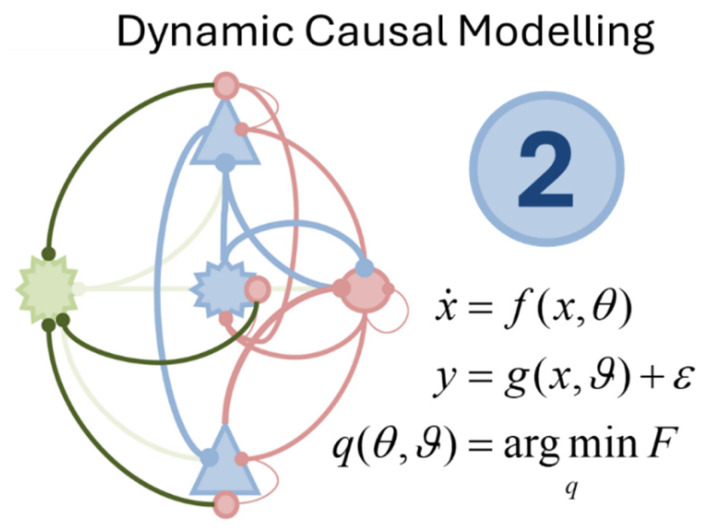
Dynamic Causal Modelling. This figure shows the second of our five formulae. This includes a pair of equations that specify a dynamic causal model, and the criterion for forming good posterior beliefs about the parameters of this model. Typically, we represent dynamics with an equation that gives the rate of change of some system variable (here, *x*)—often a vector—as a function (*f*) of the current value that variable takes. In neural systems, the *x*-vector comprises summaries of populations of neurons (shown here with excitatory neurons in blue, inhibitory in red, and green glial cells). For instance, it may include the average firing rates and membrane potentials of neurons in each brain region. The connectivity between regions can then be summarised by parameters (*θ*) that play the role of rate constants, determining the rate of change of membrane potentials in one population as a function of the firing rate of another. Such systems can be solved either analytically or, more often, numerically to give trajectories of the unobserved (hidden) variables and predictions as to the data (*y*) they generate. This figure shows a simple version of the equations for (DCM) based upon a deterministic differential equation. This equips the dynamics of the hidden states with a function (*g*) that predicts the data we might observe given that state, and with some measurement noise (*ε*). In models of electrophysiological data, the *g* function is normally referred to as a ‘lead field’ which maps from the source of a signal to its measurement—normally on the scalp surface with electro- or magnetoencephalography. In fMRI it can include the haemodynamic processes that generate a blood oxygen level dependent (BOLD) response.

**Figure 4:**
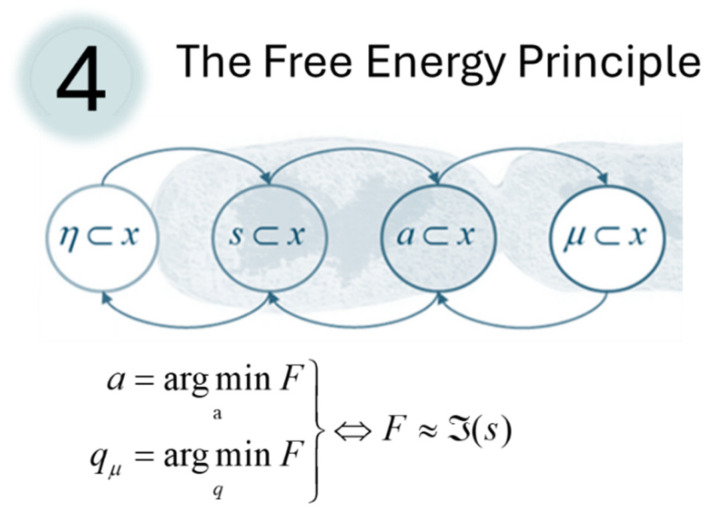
The Free Energy Principle. This figure shows the fourth of our five formulae. Here, we introduce the (Markov blanket) partition into external (*η*), sensory (*s*), active (*a*), and internal (*μ*) states. Taking the perspective of the internal and active (collectively, autonomous) states, one can interpret their dynamics as optimising a free energy functional through changing a distribution (*q*) parameterised by the internal states to fit an implicit set of beliefs to the sensory states, and through acting to change the sensory states to better fit our beliefs. When the belief distribution is optimised, free energy approximates self-information or surprise (ℑ), the negative log probability of sensory states. The graphic is adapted from [55].

**Figure 5:**
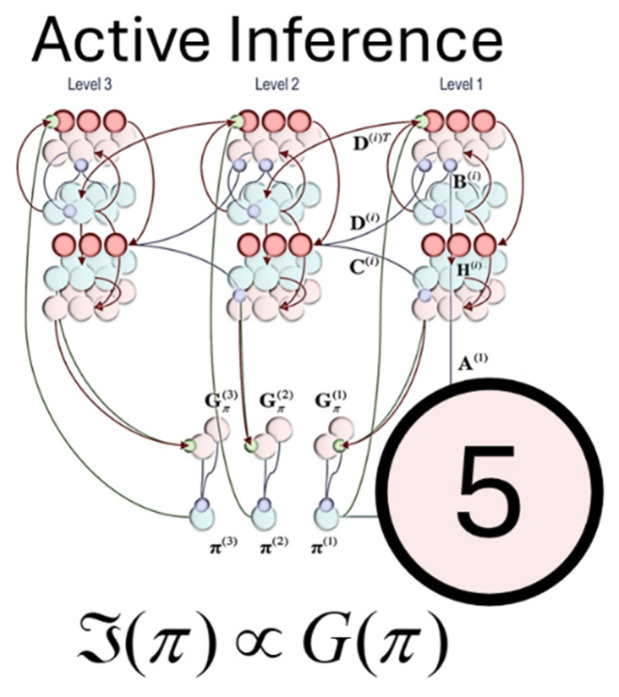
Active Inference. This figure shows the fifth of our five formulae. Here, the surprise associated with a policy (*π*) is expressed in terms of its expected free energy (*G*). Expected free energy quantifies the prior weight assigned to alternative behavioural trajectories we might select and depends upon a balance between epistemic (information seeking) and pragmatic (value seeking) terms. The associated graphic, adapted from [74] details the message passing between populations of neurons that might compute beliefs about states of the world under different behavioural trajectories and use these to determine the associated expected free energies. As in Figure 3, there is a hierarchical organisation, with stereotyped asymmetric message passing as we might expect between different cortical regions. However, each cortical layer is now equipped with a loop through subcortical structures (the lower part of the graphic) that might evaluate alternative policies.

## Data Availability

No data were used in the preparation of this article.
